# Inference from the analysis of genetic structure of *Helicobacter pylori* strains isolates from two paediatric patients with recurrent infection

**DOI:** 10.1186/s12866-019-1554-z

**Published:** 2019-08-08

**Authors:** Sandra Mendoza-Elizalde, Ana Caren Cortés-Márquez, Gerardo Zuñiga, René Cerritos, Pedro Valencia-Mayoral, Alejandra Consuelo Sánchez, Hector Olivares-Clavijo, Norma Velázquez-Guadarrama

**Affiliations:** 1 Infectology Laboratory, Hospital Infantil de México Federico Gómez, Dr. Márquez 162, Col. Doctores, Cuauhtémoc, 06720 Mexico City, Mexico; 2 Biological Chemistry Sciences Postgraduate, Escuela Nacional de Ciencias Biológicas, Instituto Politécnico Nacional, Mexico City, Mexico; 3 Biological Variation and Evolution Laboratory, Department of Zoology, Escuela Nacional de Ciencias Biológicas, Instituto Politécnico Nacional, Mexico City, Mexico; 4 Center of Research in Population and Health Policy, UNAM, Mexico City, Mexico; 5 Department of Pathology, Hospital Infantil de México Federico Gómez, Mexico City, Mexico; 6 Department of Gastroenterology and Nutrition, Hospital Infantil de México Federico Gómez, Mexico City, Mexico; 7 Hemerobiblioteca, Hospital Infantil de México Federico Gómez, México City, Mexico

**Keywords:** *Helicobacter pylori*, Recurrent infection, Reinfection, Paedriatric patients, Genetic variability, Evolutionary relationship

## Abstract

**Background:**

*Helicobacter pylori* recurrence after successful eradication is an important problem. Children are particularly vulnerable to reinfection, by intrafamilial transmission which facilitates the acquisition or recombination of new genetic information by this bacterium. We investigated the evolutionary dynamics of 80 *H. pylori* strains isolated from two paediatric patients with recurrent infection (recrudescence and reinfection).

**Results:**

We characterized the virulence genes *vacA* (*s1*, *m1*, *s2*, and *m2*), *cagA*, *cagE*, and *babA2* and performed multilocus sequence typing (MLST) on 7 housekeeping genes (*atpA*, *efp*, *ureI*, *ppa*, *mutY*, *trpC*, and *yphC*) to infer the evolutionary dynamics of the *H. pylori* strains through phylogenetic and genealogic inference analyses, genetic diversity analysis and the exploration of recombination events during recurrent infections. The virulence genotype *vacAs1m1/cagA+/cagE+/babA2* was present at a high frequency, as were the EPIYA motifs EPIYA-A, −B and -C. Furthermore, the housekeeping genes of the *H. pylori* strains exhibited high genetic variation, comprising 26 new alleles and 17 new Sequence Type (ST). In addition, the hpEurope (76.5%) and hspWAfrica (23.5%) populations predominated among the paediatric strains. All strains, regardless of their ancestral affiliation, harboured western EPIYA motifs.

**Conclusions:**

This study provides evidence of the evolutionary dynamics of the *H. pylori* strains in two paediatric patients during recrudescence and reinfection events. In particular, our study shows that the strains changed during these events, as evidenced by the presence of different STs that emerged before and after treatment; these changes may be due to the accumulation of mutations and recombination events during the diversification process and recolonization of the patients by different genotypes.

**Electronic supplementary material:**

The online version of this article (10.1186/s12866-019-1554-z) contains supplementary material, which is available to authorized users.

## Background

*Helicobacter pylori* is a gram-negative, pleomorphic, microaerophilic bacterium that has adapted to survive the extreme conditions of the human stomach [1]. Infection by this bacterium generally occurs during childhood and persists throughout the life of the host if it is not eradicated [2]. The infection is mostly asymptomatic and, to a lesser extent, is associated with chronic gastritis, gastroduodenal ulcers, mucosa-associated lymphoid tissue lymphoma and gastric cancer [3, 4].

Generally, bacteria exhibit clonal propagation during infection; however, *H. pylori* increases its adaptation potential by being highly recombinant [5]. The transmission from parents to offspring [6–8], horizontal gene transfer occurs through contaminated food, water, and non-parental caregivers [9], facilitates the acquisition or recombination of new genetic information by this specie.

*H. pylori* is an example of successful bacterial adaptation because it has evolved with its host [10, 11]. Phylogeographic studies performed with multilocus sequence typing (MLST) have shown that the dispersal of *H. pylori* throughout the world is associated with human evolution because *H. pylori* has infected humans since before the migration from Africa occurred [12]. This evolution of *H. pylori* is observed in the different recorded molecular variants associated with different geographical areas [11, 13–16].

One of the evolutionary features of *H. pylori* is its great capacity for recombination during infection in humans. A study performed to determine the sequence type (ST) diversification of *H. pylori* strains in an animal model showed that in only three months, the original strain ST181 accumulated both synonymous and non-synonymous mutations in different housekeeping genes, giving rise to new STs (ST2902 and ST2903) [17]. In addition, several changes occurred in the strains through allelic recombination in the *cagA* and *babA*2 genes, and nucleotide substitutions in the *vacA* gene, as well as through the development of chimeric *vacAs2m1* strains, thus confirming that genetic variation, which is critical for adaptation to specific conditions or environments within the host [17].

Studies in humans have revealed the presence of dominant strains; however, most studies use only one isolate per patient, resulting in very few reports of mixed infections. In a previous study, we characterized the virulence genes *cagA, cagE, vacA* and *babA2* in five isolates from each patient analyzed, and our results showed that 75% of patients had mixed infections, some with up to 5 different genotypes [18]. Other studies that have sequenced the complete genome have also found different genotypes apparently produced by recombination [19]. In contrast, studies employing MLST have detected patients infected with strains containing the same STs or with closely related strains [9, 20].

During recurrent *H. pylori* infection, even when patients are treated, signs and symptoms of disease reappear, resulting in two clinically important mechanisms: recrudescence and reinfection. Recrudescence is defined as the apparent elimination of infection due to bacterial suppression but not eradication [21, 22], while reinfection occurs when a patient is reinfected after successful eradication with a new strain or with the same strain at least one year after eradication [23, 24]. Both mechanisms of infection by *H. pylori* assume that the bacterial population is suppressed whether or not the treatment used is effective, thus affecting the clinical diagnosis.

The mechanisms of recurrence have effects on the *H. pylori* population before and after clinical treatment because the population can experience contraction and expansion events of genetic variation due to the reduction and increase in the population size. Therefore, to infer the evolutionary dynamics of *H. pylori*, we performed phylogenetic and genealogy inference analyses, genetic diversity analysis, and the exploration of possible recombination events in 80 *H. pylori* strains isolated from two paediatric patients with recurrent infection. The *H. pylori* strains were characterized before and after treatment in these patients, who were diagnosed by their symptoms as experiencing recrudescence or reinfection. In particular, we were interested in 1) determining whether the suppressed strains in the host are the same over time (recrudescence) and 2) determining whether the reinfection occurred with different strains.

## Results

The analysis of 80 strains isolated from two paediatric patients with recurrent *H. pylori* infection (40 strains per patient), showed the antimicrobial susceptibility profile. In patient one, 100% of the strains involved in the first event were sensitive to CLA (0.015 μg/mL) and AMX (0.25 μg/mL) but resistant to MTZ (16 μg/mL); during the second event, the MICs of the strains were different but they did not show changes in the sensitivity, remaining sensitive to CLA (0.015–0.031 μg/mL) and AMX (0.015 μg/mL) but resistant to MTZ (32–128 μg/mL). Similarly, in patient two, 100% of the strains involved in the first event were sensitive to CLA (< 0.0078–0.031 μg/mL) and AMX (0.015 μg/mL) but resistant to MTZ (16–32 μg/mL); during the second event, the MICs of the strains were different, without changes in the sensitivity 100% of these strains were sensitive to CLA (0.015–0.031 μg/mL) and AMX (0.0078–0.015 μg/mL) but resistant to MTZ (8–32 μg/mL) (Table 1).

**Table 1:** Susceptibility profile of the *Helicobacter pylori* strains identified from paedriatic patients during different events

Strain	CLA Event		AMX Event		MTZ Event		TC Event	
	First	Second	First	Second	First	Second	First	Second
	[μg/mL]		[μg/mL]		[μg/mL]		[μg/mL]	
Patient One								
1	0.015	0.031	0.25	0.015	16	64	0.125	0.062
2	0.015	0.015	0.25	0.015	16	64	0.5	0.062
3	0.015	0.031	0.25	0.015	16	32	0.5	0.062
4	0.015	0.015	0.25	0.015	16	64	0.5	0.062
5	0.015	0.015	0.25	0.015	16	128	0.25	0.062
6	0.015	0.031	0.25	0.015	16	64	0.25	0.062
7	0.015	0.015	0.25	0.015	16	64	0.5	0.062
8	0.015	0.015	0.25	0.015	16	32	0.25	0.062
9	0.015	0.015	0.25	0.015	16	64	0.25	0.062
10	0.015	0.015	0.25	0.015	16	128	0.125	0.062
11	0.015	0.031	0.25	0.015	16	64	0.5	0.062
12	0.015	0.015	0.25	0.015	16	64	0.5	0.062
13	0.015	0.031	0.25	0.015	16	32	0.5	0.062
14	0.015	0.015	0.25	0.015	16	64	0.25	0.062
15	0.015	0.015	0.25	0.015	16	128	0.25	0.062
16	0.015	0.015	0.25	0.015	16	64	0.5	0.062
17	0.015	0.015	0.25	0.015	16	64	0.25	0.062
18	0.015	0.031	0.25	0.015	16	64	0.25	0.062
19	0.015	0.015	0.25	0.015	16	128	0.5	0.062
20	0.015	0.015	0.25	0.015	16	64	0.25	0.062
Patient Two								
1	< 0.078	0.031	0.015	0.0078	32	8	0.062	0.062
2	< 0.078	0.015	0.015	0.062	32	8	0.062	0.25
3	< 0.078	0.031	0.015	0.015	32	16	0.031	0.062
4	0.031	0.031	0.015	0.0078	16	32	0.062	0.062
5	< 0.078	0.031	0.015	0.031	64	8	0.062	0.062
6	< 0.078	0.015	0.015	0.062	32	8	0.062	0.25
7	< 0.078	0.015	0.015	0.062	32	8	0.031	0.062
8	< 0.078	0.031	0.015	0.015	32	16	0.062	0.062
9	< 0.078	0.015	0.015	0.062	32	8	0.062	0.25
10	< 0.078	0.031	0.015	0.015	32	16	0.031	0.062
11	0.031	0.031	0.015	0.0078	16	32	0.062	0.062
12	< 0.078	0.031	0.015	0.031	64	8	0.062	0.062
13	< 0.078	0.015	0.015	0.062	32	8	0.062	0.25
14	< 0.078	0.031	0.015	0.015	32	16	0.031	0.062
15	0.031	0.031	0.015	0.0078	16	32	0.062	0.062
16	< 0.078	0.031	0.015	0.031	64	8	0.125	0.031
17	< 0.078	0.015	0.015	0.062	32	8	0.062	0.25
18	< 0.078	0.031	0.015	0.015	32	16	0.031	0.062
19	0.031	0.031	0.015	0.0078	16	32	0.062	0.062
20	< 0.078	0.031	0.015	0.031	64	8	0.062	0.25

CLA: Clarithromycin (Resistant ≥1 μg/mL CLSI 2015) AMX: Amoxicillin (Resistant ≥4 g/mL Torres et al., 2001) MTZ: Metronidazole (Sensitive < 2 μg/mL; Intermediate 2 to 4 μg/mL; Low Resistant 8 to 16 μg/mL, Moderate Resistant 32 to 64 μg/mL; High Resistant ≥128 μg/mL Poon et al., 2009; Maggi et al., 2000 TC: Tetracycline ≥4 μg/mL Gerrits et al., 2003)

Likewise, the 80 strains exhibited variable frequencies of the virulence genes within the mosaic *vacA* (*s1*, *s2*, *m1*, and *m2*) structure (Tables 2 and 3). For *vacA*, allele *s1* was the most frequent, at 77.5% (62/80), while alleles *s2*, at 20% (16/80), and *m2*, at 1% (1/80), were the least frequent. The frequencies of the *cagA* and *cagE* genes, which belong to the *cag*-PAI (Pathogenicity Island), were 97.5% (78/80) and 98.75% (79/80), respectively. The *babA2* gene was present in 25% (20/80) of strains. The following EPIYA motifs were identified in the polymorphic region of the CagA protein: 41.25% (33/80) type ABC, 26.25% (21/80) type ABCC, 26.25% (21/80) type ABCCC, 1.25% (1/80) type AAB^&^C, 1.25% (1/80) type AB^&^C and 1.25% (1/80) type ABC^&^ (Additional file 1: Table S1). Six different genotypes were identified among the paediatric strains.

**Table 2:** Characteristics of the *Helicobacter pylori* strains identified from patient one during different events

Patient one				
Date of biopsy	First event			
	Strain	ST	Virulence genes	**EPIYA motif**
August 2006	1	2888	*vacAs1+ / cagA+ / cagE+ / babA2+*	ABC
	2	2889	*vacAs1m1 / cagA+ / cagE+ / babA2-*	ABCC
	3	2890	*vacAs1m1 / cagA+ / cagE+ / babA2-*	ABCC
	4	313	*vacAs2m1 / cagA+ / cagE+ / babA2-*	ABCC
	5	313	*vacAs1m1 / cagA+ / cagE+ / babA2-*	ABCC
	6	2891	*vacAs1m1 / cagA+ / cagE+ / babA2-*	ABCCC
	7	288	*vacAs1m1 / cagA+ / cagE+ / babA2-*	ABCC
	8	313	*vacAs1m1 / cagA+ / cagE+ / babA2-*	ABCC
	9	288	*vacAs1m1 / cagA+ / cagE+ / babA2-*	ABCC
	10	313	*vacAs1m1 / cagA+ / cagE+ / babA2-*	ABCC
	11	313	*vacAs1m1 / cagA+ / cagE+ / babA2-*	ABCC
	12	313	*vacAs1m1 / cagA+ / cagE+ / babA2-*	ABCC
	13	313	*vacAs1m1 / cagA+ / cagE+ / babA2-*	ABCC
	14	313	*vacAs1m1 / cagA+ / cagE+ / babA2-*	ABCC
	15	313	*vacAm1+ / cagA+ / cagE+ / babA2-*	ABCC
	16	2892	*vacAs2m1 / cagA+ / cagE+ / babA2-*	ABCC
	17	313	*vacAs2m1 / cagA+ / cagE+ / babA2-*	ABCC
	18	313	*vacAs2m1 / cagA+ / cagE+ / babA2-*	ABCC
	19	2893	*vacAs2m1 / cagA+ / cagE+ / babA2-*	ABCC
	20	313	*vacAs2m1 / cagA+ / cagE+ / babA2-*	ABCC
October 2007	Second event			
	Strain	ST	Virulence genes	EPIYA motif
	1	288	*vacAs2m1 / cagA+ / cagE+ / babA2-*	ABCCC
	2	288	*vacAs1m1 / cagA+ / cagE+ / babA2-*	ABCCC
	3	288	*vacAs1m1 / cagA+ / cagE+ / babA2-*	ABCCC
	4	288	*vacAs1m1 / cagA+ / cagE+ / babA2-*	ABCCC
	5	313	*vacAs1m1 / cagA+ / cagE+ / babA2-*	ABCCC
	6	288	*vacAs1m1 / cagA+ / cagE+ / babA2-*	ABCCC
	7	288	*vacAs1m1 / cagA+ / cagE+ / babA2-*	ABCCC
	8	288	*vacA- / cagA+ / cagE+ / babA2-*	ABCCC
	9	288	*vacAs1m1 / cagA+ / cagE+ / babA2-*	ABCC
	10	288	*vacAs1m1 / cagA+ / cagE+ / babA2-*	ABCCC
	11	288	*vacAs1m1 / cagA+ / cagE+ / babA2-*	ABCCC
	12	288	*vacAs1m1 / cagA+ / cagE+ / babA2-*	ABCCC
	13	813	*vacAs1m1 / cagA+ / cagE+ / babA2-*	ABCCC
	14	313	*vacAs1m1 / cagA+ / cagE+ / babA2-*	ABCCC
	15	288	*vacAs1m1 / cagA+ / cagE+ / babA2-*	ABCCC
	16	288	*vacAs2m1 / cagA+ / cagE+ / babA2-*	ABCCC
	17	288	*vacAs2m1 / cagA+ / cagE+ / babA2-*	ABCCC
	18	2887	*vacAs2m1 / cagA+ / cagE+ / babA2-*	ABCCC
	19	313	*vacAs1m1 / cagA+ / cagE+ / babA2-*	ABCCC
	20	288	*vacAs2m1 / cagA+ / cagE+ / babA2-*	ABCCC

ST: Sequence Type

**Table 3:** Characteristics of the *Helicobacter pylori* strains identified from patient two during different events

Patient two				
Date of biopsy	First event			
	Strain	ST	Virulence genes	EPIYA motif
October 2007	1	2894	*vacAs1m1 / cagA+ / cagE+ / babA2-*	ABC^&^
	2	2895	*vacAs1m1 / cagA+ / cagE+ / babA2+*	ABC
	3	2896	*vacAs1m1 / cagA+ / cagE+ / babA2+*	AAB^&^C
	4	2894	*vacAs1m1 / cagA+ / cagE+ / babA2-*	ABC
	5	2894	*vacAs1m1 / cagA+ / cagE+ / babA2-*	ABCC
	6	2894	*vacAs1m1 / cagA+ / cagE+ / babA2+*	ABCC
	7	2894	*vacAs1m1 / cagA+ / cagE+ / babA2-*	ABC
	8	2894	*vacAs1m1 / cagA+ / cagE+ / babA2-*	ABC
	9	2894	*vacAs1m1 / cagA+ / cagE+ / babA2-*	ABC
	10	2894	*vacAs1m1 / cagA- / cagE+ / babA2+*	NEGATIVE
	11	2894	*vacAs1m1 / cagA+ / cagE+ / babA2+*	ABC
	12	2897	*vacAs1+ / cagA+ / cagE- / babA2-*	ABC
	13	2894	*vacAs1m1 / cagA+ / cagE+ / babA2-*	ABC
	14	2894	*vacAs1m1 / cagA+ / cagE+ / babA2+*	ABC
	15	2898	*vacAs1m1 / cagA+ / cagE+ / babA2-*	ABC
	16	2894	*vacAs1m1 / cagA+ / cagE+ / babA2-*	ABC
	17	2894	*vacAs1m1 / cagA+ / cagE+ / babA2-*	ABC
	18	2894	*vacAs1m1 / cagA+ / cagE+ / babA2-*	ABC
	19	2898	*vacAs1m1 / cagA+ / cagE+ / babA2-*	ABC
	20	2898	*vacAs1m1 / cagA+ / cagE+ / babA2-*	ABC
June 2008	Second event			
	Strain	ST	Virulence genes	EPIYA motif
	1	2894	*vacAs1m1 / cagA+ / cagE+ / babA2-*	ABC
	2	2894	*vacAs1m1 / cagA+ / cagE+ / babA2+*	AB^&^C
	3	2894	*vacAs1m1 / cagA+ / cagE+ / babA2+*	ABC
	4	2894	*vacAs1m1 / cagA+ / cagE+ / babA2+*	ABC
	5	2894	*vacAs1m1 / cagA+ / cagE+ / babA2-*	ABC
	6	2894	*vacAs1m1 / cagA+ / cagE+ / babA2+*	ABC
	7	2894	*vacAs2m1 / cagA+ / cagE+ / babA2+*	ABC
	8	2894	*vacAs1m1 / cagA+ / cagE+ / babA2+*	ABC
	9	2894	*vacAs1m1 / cagA+ / cagE+ / babA2+*	ABC
	10	2894	*vacAs1m1 / cagA+ / cagE+ / babA2+*	ABC
	11	2894	*vacAs1m1 / cagA- / cagE+ / babA2-*	NEGATIVE
	12	2894	*vacAs1m1 / cagA+ / cagE+ / babA2+*	ABC
	13	2899	*vacAs2m1 / cagA+ / cagE+ / babA2+*	ABC
	14	2894	*vacAs2m1 / cagA+ / cagE+ / babA2+*	ABC
	15	2894	*vacAs2m1 / cagA+ / cagE+ / babA2+*	ABC
	16	2900	*vacAs1m1 / cagA+ / cagE+ / babA2-*	ABC
	17	2894	*vacAs2m1 / cagA+ / cagE+ / babA2+*	ABC
	18	2894	*vacAs1m1 / cagA+ / cagE+ / babA2-*	ABC
	19	2894	*vacAs1m1 / cagA+ / cagE+ / babA2-*	ABC
	20	2894	*vacAs1m1 / cagA+ / cagE+ / babA2-*	ABCCC

ST: Sequence Type; &: Amino acid change in EPIYA motif

During the first event, patient one harboured the genotypes *vacAs1m1*/*cagA*+/*cagE*+/*babA2*- and *vacAs2m1*/*cagA*+/*cagE*+/*babA2*- in 12/20 and 6/20 strains, respectively. In addition, this patient had a greater number of strains containing the EPIYA-ABCC motif (18/20) than the other motifs. The same genotypes were present during the second event (14/20 and 5/20 strains, respectively), although a higher number of strains containing the EPIYA-ABCCC motif (19/20) was observed (Table 2). Patient two harboured the genotypes *vacAs1m1/cagA*+/*cagE*+/*babA2*-, *vacAs1m1*/*cagA*+/*cagE*+/*babA2*+ and *vacAs1m1*/*cagA−*/*cagE*+/*babA2*+ in 13/20, 5/20 and 1/20 strains, respectively, during the first event. In addition, patient two harboured greater numbers of strains containing the EPIYA-ABC motif (15/20) and the EPIYA-ABC^&^ and EPIYA-AAB^&^C motifs. The genotypes *vacAs1m1/cagA+/cagE+/babA2-, vacAs1m1/cagA+/cagE+/babA2+, vacAs2m1/cagA+/cagE+/babA2+* and *vacAs1m1/cagA−/cagE+/babA2-* (6/20, 8/20, 5/20 and 1/20 strains, respectively) were present during the second event. In addition, there were more EPIYA-ABC (17/20) and EPIYA-AB^&^C motifs during the second event (Table 3).

The analysis of seven housekeeping genes in the *H. pylori* strains revealed 26 new alleles (*atpA*, 4 alleles; *efp*, 3 alleles; *mutY*, 5 alleles; *ppa*, 2 alleles; *trpC*, 4 alleles; *ureI*, 4 alleles; and *yphC*, 4 alleles) (Additional file 2: Table S2). The genetic diversity, as measured by the π and θ indices, was high in all genes, with *trpC* in the first event in both patients presenting the greatest diversity. In most of the analysed genes, it was found that the θ values were higher than the π values, which indicates that there are some haplotypes that are very divergent. The number of haplotypes at each locus ranged from one to four (Table 4).

**Table 4:** Polymorphisms and features of housekeeping genes of *Helicobacter pylori* strains obtained from paediatric patients

	Gene	First event					Second event				
		Polymorphic Sites	Haplotype	Hd	Π	Θ	Polymorphic Sites	Haplotype	Hd	Π	Θ
Patient one	*atpA*	23	2	0.100	0.0036	0.0104	0	1	0	0	0
	*efp*	17	3	0.279	0.0087	0.0116	0	1	0	0	0
	*mutY*	32	3	0.195	0.0084	0.0214	0	1	0	0	0
	*ppa*	10	2	0.189	0.0047	0.0070	20	3	0.353	0.00960	0.01416
	*trpC*	45	4	0.284	0.0124	0.0290	0	1	0	0	0
	*ureI*	31	4	0.284	0.0081	0.0159	14	2	0.100	0.00239	0.00675
	*yphC*	0	1	0.000	0	0	0	1	0	0	0
	Concatenated	158	8	0.647	0.00640	0.0134	34	4	0.432	0.00153	0.00281
Patient two	*atpA*	17	3	0.195	0.00357	0.0076	23	2	0.100	0.00367	0.1034
	*efp*	9	2	0.100	0.00220	0.0061	9	2	0.100	0.00220	0.00619
	*mutY*	37	3	0.195	0.01051	0.0248	0	1	0	0	0
	*ppa*	18	2	0.100	0.00452	0.0127	0	1	0	0	0
	*trpC*	50	3	0.195	0.01412	0.0315	0	1	0	0	0
	*ureI*	18	2	0.100	0.00308	0.0086	18	2	0.100	0.00308	0.00867
	*yphC*	19	3	0.353	0.00406	0.1050	21	2	0.100	0.00412	0.01161
	Concatenated	168	5	0.505	0.00577	0.0139	71	3	0.195	0.00208	0.00588

Π: Nucleotide diversity per site; Θ: Average number of nucleotide differences per site

Both paediatric patients demonstrated infection recurrence with 17 new ST sequences. In patient one, ST313 (12/20) predominated in the first infection event, followed by ST288, ST2888, ST2889, ST2890, ST2891, ST2892 and ST2893. ST288 (15/20) predominated during the second infection event, and two new STs, ST813 and ST2887, were present (Table 2). In patient two, we identified ST2894, ST2895, ST2896, ST2897 and ST2898 during the first infection event, while ST2894 prevailed in 18 strains; two new STs, ST2899 and ST2900, emerged during the second infection event (Table 3).

**Fig. 1:**
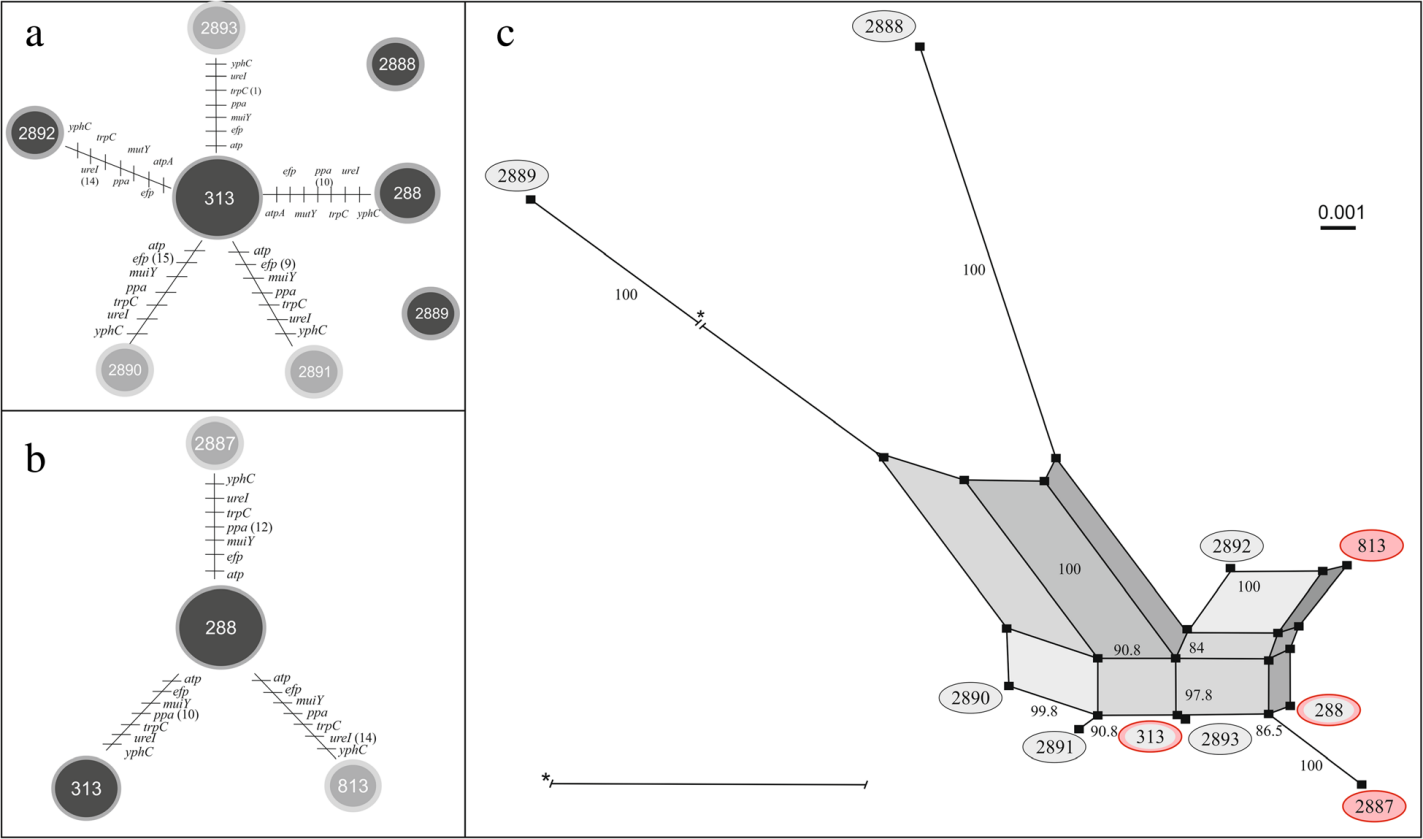
Evolutionary history among the STs of *Helicobacter pylori* identified in patient one with recurrent infection. **a** and **b** show the clonal relationships among the STs of *H. pylori* during the first and second infection events, respectively. Each line represents a different allele with mutational changes. PHYLOViZ (goeBURST algorithm) was used to define the clonal relationships [25]. **a** The main clonal complex in the first event was ST313 (12 strains), with five linked STs and two unlinked STs. **b** The main clonal complex in the second event was ST288 (15 strains), with three STs. c) Evolutionary relationships among the STs of *H. pylori* during both events. The neighbour-net graph defines the evolutionary relationships [26]; the black circles indicate the STs identified during the first infection event, and the red circles indicate the STs identified during the second infection event. Bootstrap values > 84% are indicated on the paths in the network. The highly branched network structure is indicative of possible recombination events among the STs

**Fig. 2:**
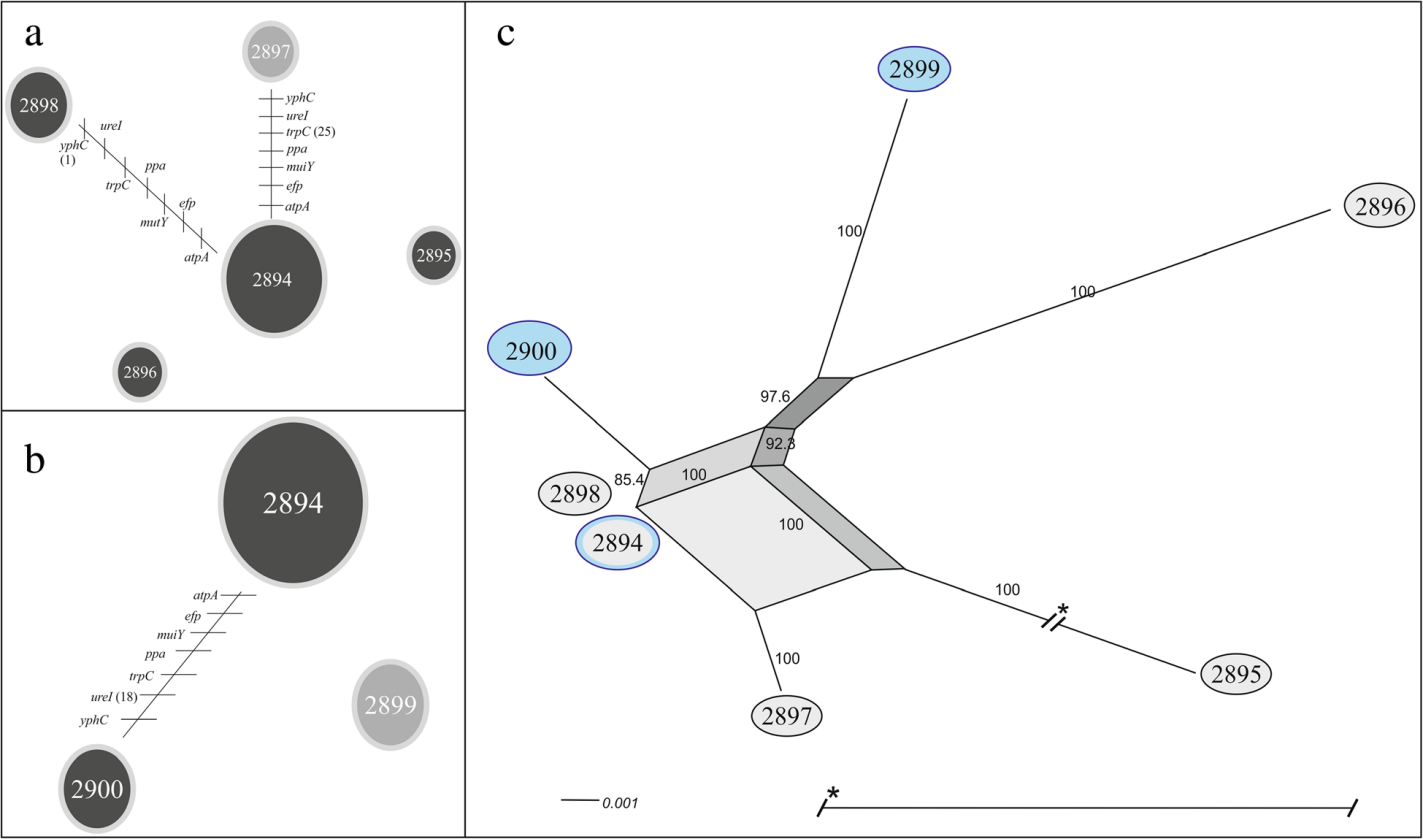
Evolutionary history among the STs of *Helicobacter pylori* identified in patient two with recurrent infection. **a** and **b** show the clonal relationships among the STs of *H. pylori* during the first and second infection events, respectively. Each line represents a different allele with mutational changes. PHYLOViZ (goeBURST algorithm) was used to define the clonal relationships [25]. **a** The main clonal complex in the first event was ST2894 (14 strains), with two linked STs and two unlinked STs. **b** The main clonal complexes in the second event were again ST2894 (18 strains), with only one ST, as well as ST2899, which was unlinked. c) Evolutionary relationships among the STs of *H. pylori* during both events. The neighbour-net graph defines the evolutionary relationships [26]; the black circles indicate the STs identified during the first infection event, and the blue circles indicate the STs identified during the second infection event. Bootstrap values > 85% are indicated on the paths in the network. The highly branched network structure is indicative of possible recombination events among the STs

The different STs identified during each event in the two paediatric patients were used as genotyping data in the PHYLOViZ platform. The goeBURST algorithm was used at the TLV level for the dataset analysis. The most frequent alleles among the 80 paediatric strains were *atpA* 2336 (49.38%), *efp* 901 (48.15%), *mutY* 2341 (46.91%) *ppa* 2199 (74.07%), *trpC* 2371 and 2413 (46.91% each), *ureI* 2386 (50%), and *yphC* 2590 (50.62%). Patient one demonstrated one genotypic signature including five (ST288, ST2890, ST2891, ST2892, ST2893) and three (ST313, ST813, ST2887) linked STs derived from the first and second infection events, respectively (Fig. 1a, b). Patient two demonstrated one genotypic signature comprising two (ST2897, ST2898) and one (ST2900) linked STs derived from the first and second event, respectively, as well as individual unlinked STs (Fig. 2a, b). The genealogy of the *H. pylori* strains in each paediatric patient was determined using the neighbour-net algorithm and showed recombinant ST networks in both patient one (Fig. 1c) and patient two (Fig. 2c) during each infection event, with bootstrap values of > 84 and 85% for patient one and patient two, respectively. In addition, the STs identified during the second event in each patient were related to those identified in the first event, because they occurred on the same side of the network. In patient one, ST288 (17/40 strains) was the most frequent, followed by ST313 (15/40 strains); ST813, ST2887, ST2888, ST2889, ST2890, ST2891, ST2892, and ST2893 were each present in only a single strain. In patient two, ST2894 (32/40 strains) was the most frequent, followed by ST2898 (3/40 strains); ST2895, ST2896, ST2897, ST2899, and ST2900 were each present in only a single strain.

**Fig. 3:**
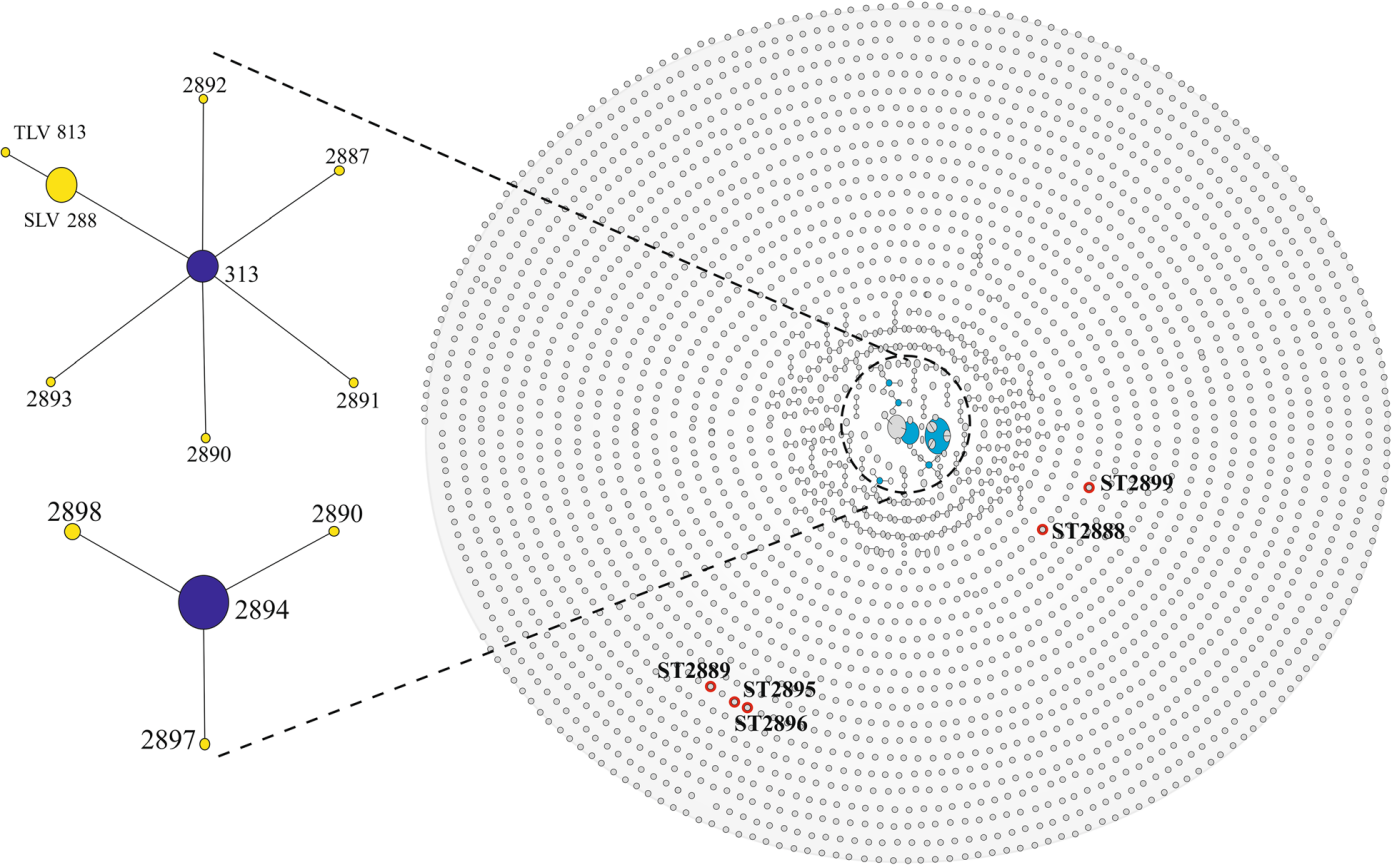
Population “snapshot” of *Helicobacter pylori* STs found in the paediatric strains and clonal complexes ST313 and ST2894. Clusters of related isolates and individual unlinked STs found in the MLST database for *H. pylori* are presented as a single eBURST tree [http://www.phyloviz.net/goeburst/] defining category zero and comprising seven shared alleles. Unions link isolates that correspond to clonal complexes. Primary founders (blue) are located in the centre of the group, and the founders of the subgroups are shown for ST2888, ST2889, ST2895, ST2896 and ST2899 (red circles); the labels for the other STs [http://pubmlst.org//helicobacter/] have been removed for clarity. The predicted primary founders are ST313 and ST2894 (bootstrap value: 1000). The primary founders (blue) are located in the centre of the group, and the founders of the subgroups are shown in yellow

Our eBURST findings provided an overview of the different clonal complexes. Clusters of related isolates and individual unlinked STs are shown as a tree, defining category zero for the seven shared alleles. The central part contains the major clonal complexes, the linked triples and doubles, and the following individual unlinked STs: ST2888, ST2889, ST2895, ST2896 and ST2899 (individually isolated) (Fig. 3). The ST313 and ST2894 clonal complexes (15/80 and 32/80 isolates, respectively) (Fig. 3) were the main founders (blue) (bootstraps: 1000). These complexes (ST313 and ST2894) were present in at least six alleles that also contained individual STs. The ST313 clonal complex contained six SLVs (yellow): ST288 (17/80 isolates) and ST2887, ST2890, ST2891, ST2892 and ST2893 (1/80 isolates each). In addition, in patient one, the SLV288 clonal complex contained TLV813 (1/80 isolates), along with the unlinked STs ST2888 and ST2889. The ST2894 clonal complex was found in patient two, and it contained only three SLVs: ST2898 (3/80 isolates), ST2897 and ST2900 (1/80 isolates each), as well as the unlinked STs ST2895, ST2896 and ST2899. Importantly, the spacing between the unlinked STs and the clonal complexes provides no information about genetic distances.

**Fig. 4:**
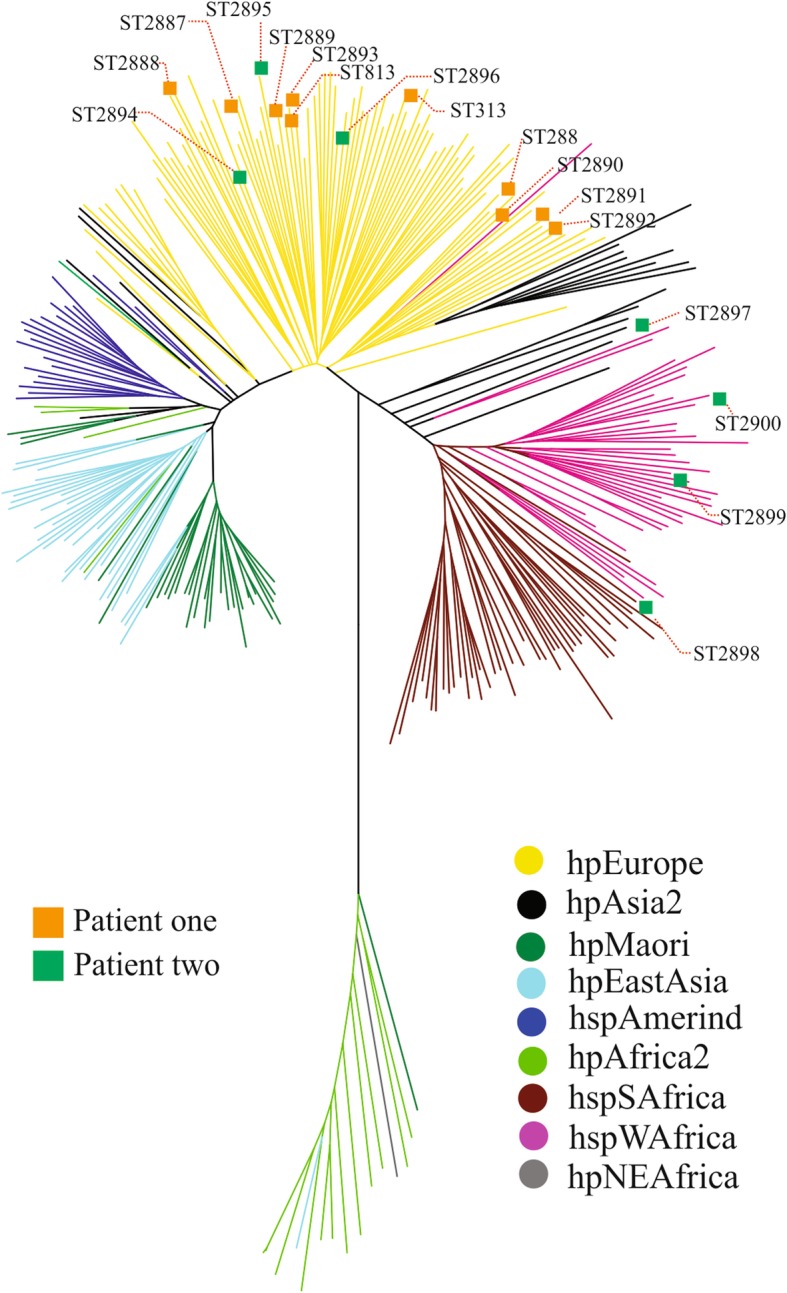
Phylogeography of the analysed STs. The phylogeography was inferred using the neighbour-joining method [27]. The optimal tree with a summed branch length of 3.97653233 is shown. The tree is drawn to scale; branch lengths with the same units as the evolutionary distances are used to infer the phylogenetic tree. The evolutionary distances were computed using the Kimura 2-parameter method [28], and the units are the number of base substitutions per site. The rate variations among the sites were modeled with a gamma distribution (shape parameter = 1). The analysis included 316 reference STs from the *H. pylori* MLST database [http://pubmlst.org/helicobacter/]. All ambiguous positions were removed for each sequence pair. The major *H. pylori* populations were identified according to the assigned population available at PubMLST and the identification and positions of the 17 STs identified in this study are shown in the tree. There were 3402 positions in the final dataset. Phylogeography analyses were conducted using MEGA6 [29]

Phylogenetic analyses of the concatenated housekeeping genes showed that the STs in this study clustered in the hpEurope population (76.5%), as did STs from other Latin American countries (Fig. 4). However, the STs also clustered within the hspWAfrica subpopulation (23.5%). In addition, all STs from patient one were grouped in the hpEurope population, while the STs from patient two were distributed between the hpEurope and hspWAfrica populations.

## Discussion

*H. pylori* is a bacterium that is acquired at an early age, and mixed infections have been reported [18, 27, 29]. *H. pylori* recurrence after successful eradication is an infrequent event and usually involves: reinfection and recrudescence. A higher recurrence of *H. pylori* infection has been observed in Latin America than in other regions of the world [28, 30, 31]. In this study, *H. pylori* strains from two Mexican paediatric patients with recurrent infection were analysed.

Patient one, who was diagnosed with reinfection, harboured the same genotypes during both events (2006–2007), suggesting that the reinfection was attributable to recrudescence and not reinfection (Table 2). The presence of identical genotypes at different times indicates treatment failure: the bacterium was suppressed but not eradicated. Xia et al. [32] reported the unsuitability of certain antimicrobial therapies for *H. pylori* because they do not completely eradicate infection, thus resulting in recurrence. The presence of antibiotic-resistant strains is one of the most frequent causes of recurrent *H. pylori* infection; in this situation, selection pressure is exerted, resulting in the death of sensitive strains and the persistence of resistant strains. However, data shown in the Table 1 indicate that strains isolated during both events did not demonstrate resistance to antibiotic treatment (AMX and CLA). Another of the most frequent causes of recrudescence is a lack of treatment adherence; this situation conditions the strains to stimuli induced by the environment, which contains different concentrations of antibiotics.

Patient two, diagnosed with recrudescence, harboured different genotypes during the second event (2008), suggesting reinfection and not recrudescence (Table 3). Reinfection is responsible for 62.5 to 75% of *H. pylori* recurrence cases in the first 6 months after eradication and is primarily attributed to intrafamilial transmission [33, 34]. The predominant genotype in both paediatric patients was *vacAs1m1/cagA+/cagE+/babA2-* (45/80 strains); however, other genotypes with different allelic combinations for the *vacA* gene were identified: *vacAs2m1* (*vacAs2m1/cagA+/cagE+/babA2* and *vacAs2m1/cagA+/cagE+/babA2+*). These allelic combinations were previously reported in paediatric strains in Mexico [17]. Importantly, the *babA2+* genotype was predominant, and this predominance is attributable to adherence to the gastric epithelium or to persistent or chronic infection [35, 36].

In addition, our findings show that the 80 strains of *H. pylori* present the EPIYA motif Western-ABC, with different repeats of EPIYA-C (41.25% ABC, 26.25% ABCC, and 26.25% ABCCC). It has been reported that in both adults and children, the prevalence of the CagA protein containing three EPIYA motifs (ABC) or four EPIYA motifs (ABCC) is the same. However, strains containing more than four EPIYA motifs (ABCCC) have also been observed in children over 10 years of age and in adults [37, 38], suggesting that CagA strains acquire more EPIYA-C repetitions over time. These results highlight the presence of EPIYA motifs in the polymorphic region of the CagA protein (Western-ABC and Eastern-ABD, the latter being more aggressive), and the number of repetitions has been associated with strains that produce greater morphological changes in gastric epithelial cells, thus resulting in an increased risk of gastric cancer [39, 40].

Strains isolated from patient one during the first event demonstrated a higher frequency of EPIYA-ABCC motifs, and strains from the second event had a higher frequency of EPIYA-ABCCC motifs (Table 2). Reyes-Leon et al. [37] reported that increases in multiple segments of the EPIYA-C motif are involved in the development of gastric diseases, and this association was observed in patient one during clinical treatment. This association was not observed in strains obtained from patient two, who presented only epigastric abdominal pain; these strains (40 isolates) presented the same frequency of the EPIYA-ABC motif during both the first and second events (Table 3). In this study, the modification of EPIYA-C motifs (DPVYA) with the ABC^&^ (1.25%) pattern and EPIYA-B motifs (EPIYT) with the AAB^&^C and AB^&^C (1.25% each) patterns was evident. In other studies, the presence of a dipeptide (ST) was observed in the sequence upstream of the EPIYA-A motif, which has already been observed in strains isolated from Greek children (GLKN [ST] EPIYAKVNKKK) [38]. The EPIYA-B motif is highly important for IL-8 secretion and cell elongation, and modifications to this motif (EPIYT) induce lower levels of cell elongation and IL-8 secretion than those induced by isolates containing normal ABC patterns [37].

We identified the presence of 26 new alleles and 17 new STs in *H. pylori*, representing the first alleles and STs reported worldwide for Mexican strains [41]. MLST analysis is a robust and consistent approach to study the ancestry and evolution of populations of *H. pylori*, which is an organism with a high degree of genetic diversity in housekeeping genes [2, 5, 12, 42]. When comparing the values of Pi and Theta, we observed that the value of Theta was higher than that of Pi, which suggests that the haplotypes forming the population in each patient are very divergent, thus confirming recolonization events (Table 4). It has been widely recognized that microorganisms tolerate a limited number of point mutations in their coding regions, and the mutations in *H. pylori* represent a possible mechanism for host adaptation [43].

Analysis of the evolutionary patterns among the *H. pylori* strains revealed one clonal complex with linked STs for each paediatric patient across both events as well as the presence of individual unlinked STs. Patient one had clonal complexes that comprised five and three linked STs for the first and second events, respectively (Fig. 1a, b). The second event (Fig. 1b) also included linked STs, confirming our genotypic observations (Table 2): the STs present were related, and the patient demonstrated recrudescence and not reinfection. Patient two had clonal complexes that comprised two and one linked STs for the first and second events, respectively, but one unlinked individual ST was present during the second event (Fig. 2b), confirming our genotypic observations, based on the emergence of a new unlinked ST, the patient presented reinfection. The reinfection of *H. pylori* is present among asymptomatic family members may facilitate the transmission within households. In addition, there is no change in the habits among the same family members (24). However, the patient two, both events recrudescence and reinfection may be involved.

The evolutionary relatedness in each paediatric patient revealed a possible recombination event between the two events (Figs. 1c and 2c). Patient one showed a greater number of recombination pathways between the two events (Fig. 1c), which explains the observed changes in the EPIYA motifs (Table 2). The presence of recombination pathways between the STs confirmed the acquisition of an EPIYA-C motif during the second event in 95% of the strains (19/20). Patient two presented several recombination pathways, based on the loss and emergence of a new ST, indicating that the strains in this patient underwent an adaptation process. Furthermore, the presence of multiple recombination pathways may contribute to the recurrence of infection (reinfection or recrudescence) observed in each patient. The presence of one or more dominant strains suggests an important natural diversification process in *H. pylori* strains over time, mainly via point mutations and inter-strain recombination events during mixed infections [5, 44, 45], which are regulated by natural selection favouring the presence of certain genotypes [17–19, 29, 46]. Antibiotic administration is a strong selective pressure that inhibits certain strains more than others or eradicates specific genotypes, thus changing the strain distribution in the host [46].

The phylogenetic relationships between the paediatric strains and the strains deposited in the PubMLST database for *H. pylori* (Fig. 3) were consistent with those observed in previous studies of *H. pylori.* This bacterium forms non-clonal populations because it has a high rate of mutation that generates a large number of alleles and a high rate of allelic recombination [5, 47, 48]. Our analysis confirmed the offspring patterns obtained using PHYLOViZ and the neighbour-net algorithm, indicating that all isolates in the same patient were genetically related and therefore derived from a common ancestor (Fig. 3) [49].

MLST analyses of seven concatenated housekeeping genes revealed a clear grouping of the various *H. pylori* strains according to different geographical regions. STs obtained in this study were clustered within the hpEurope group (76.5%) and the hspWAfrica subgroup (23.5%) (Fig. 4). The migrations of slaves from West Africa to the Americas and of European colonists to the Americas and South Africa are likely responsible for the current existence of these strains in Mexico as well as the incorporation of other Latin American strains into this group and subgroup [42, 50]. In addition, intra-genomic and inter-genomic diversity potentially play important roles in the presence of our strains within these groups. Last, our studies suggest that the strains isolated from patients one and two appear to possess the genetic diversity necessary to survive in the host, thus resulting in competition between genotypes during colonization.

## Conclusions

The prevalence of *H. pylori* infection depends on several important elements such as host factors, environmental factors, and genetic variation of the strains [51], thus resulting in multiple infections, convergent mutations, and recombination among strains of *H. pylori* [52–54]. This study provides evidence of the evolutionary dynamics of the *H. pylori* strains in two paediatric patients during recrudescence and reinfection events. In particular, our study shows the presence of different STs that emerged before and after treatment; these changes may be due to the accumulation of mutations and recombination events during the diversification process and recolonization of the patients by different genotypes.

## Methods

### Patients and strains

The two paediatric patients from the Department of Gastroenterology and Nutrition of the Hospital Infantil de Mexico Federico Gómez with recurrent *H. pylori* infection were both diagnosed with dyspepsia and gastroesophageal reflux disease via lower panendoscopy. Patient one was diagnosed in August 2006 (First event). Treatment with amoxicillin, clarithromycin and omeprazole was indicated, and he was asymptomatic for 13 months. The second event was diagnosed in this patient in October 2007; at this time, the patient exhibited sudden bleeding in the upper digestive tract. Patient two was diagnosed in October 2007 (First event). Treatment was indicated, but it was not administered. In January 2008, treatment with amoxicillin, clarithromycin and omeprazole was again indicated, and the symptoms disappeared two months after treatment. The second event diagnosed in this patient occurred in July 2008. Clinical diagnoses of gastritis, duodenitis, and oesophagitis were determined by endoscopic evaluation.

### Isolation and identification of *H. pylori*

Antral gastric biopsy specimens from both patients were homogenized, inoculated and cultured as described by Mendoza-Elizalde et al. [18]. Briefly, the strains were inoculated and cultured on Casman agar plates (BD BBL, MD, USA) supplemented with 5% horse blood and antibiotics under microaerophilic conditions at 37 °C for 5–7 days. Twenty strains were isolated for each infection event, for a total of 40 strains per patient. Bacterial identification was based on colony morphology, Gram staining, and tests for urease, oxidase and catalase activity. Eighty colonies isolated from the two patients were stored at − 70 °C in 1.5 mL of Brucella broth (BD BBL) supplemented with 10% foetal bovine serum and 25% glycerol.

### Susceptibility profile

The minimum inhibitory concentrations (MICs) to three different antibiotic classes, including the β-lactam amoxicillin (AMX, Sigma-Aldrich, St. Louis, MO), the macrolide clarithromycin (CLA, MP Biomedicals, Solon, OH) and the nitroimidazole metronidazole (MTZ, Sigma-Aldrich, St. Louis, MO), were performed using agar dilution methods according to the Clinical and Laboratory Standards Institute (CLSI) guidelines (2015) [55]. The reference strain used for the validation of the techniques was *Helicobacter pylori* ATCC® 43,504 (American Type Culture Collection, Manassas, VA, USA). The minimal inhibitory concentration interpretative criteria (μg/mL) for resistance was as follows: CLA: ≥1, AMX: ≥4, and MTZ: > 8 [56–59].

### Polymerase chain reaction (PCR) detection of virulence genes

Genomic DNA was extracted from cultured *H. pylori* using a Wizard Genomic DNA Purification Kit (Promega, Madison, WI, USA) according to the manufacturer’s instructions, with slightly modified incubation times. The quantity and integrity of the DNA was analyzed as described by Mendoza-Elizalde et al. [18]. *H. pylori* was identified based on the presence of the *glmM* gene [60]. The *vacA* (*s1, s2, m1,* and *m2*), *cagA, cagE*, and *babA2* genes were amplified by PCR using the conditions described by Atherton et al. [61], Mizushima et al. [62] and Kauser et al. [63]. DNA from *H. pylori* reference strain 26,695 was included as a positive control, and DNA from *Pseudomonas aeruginosa* reference strain PAO1 was included as a negative control. Amplification was performed using the conditions described by Mendoza-Elizalde et al. [18] in a T100™ Bio-Rad thermal cycler (Applied Biosystems, Foster City, CA, USA). The PCR products were separated and stained as described by Mendoza-Elizalde et al. [18].

### Amplification of the 3′ variable region of cagA

The 3′ variable region of the *cagA* gene was amplified using the conditions described by Mendoza-Elizalde et al. [18]. The primers used were those described by Rudi et al. [64]. The PCR products were separated by electrophoresis on 1.5% agarose gels. The PCR products were purified using ExoSap IT® (Affymetrix, Cleveland, OH, USA) according to the manufacturer’s recommendations. The purified products were sequenced using a BigDye Terminator v3.1 Cycle Sequencing Kit in an ABI 3130 genetic analyser (Applied Biosystems, Foster City, CA, USA). The sequences obtained were aligned using the CAP3 Sequence Assembly program (available at: http://doua.prabi.fr/softwore/cap3). After alignment, the nucleotide sequences were translated into amino acid sequences using the Blastx program (available at http://blast.ncbi.nlm.nih.gov/Blast.cgi) and compared with sequences deposited in GenBank (http://www.ncbi.nlm.nih.gov/genbank/).

### Multi-locus sequence typing (MLST)

Seven housekeeping genes of *H. pylori* located throughout the genome were amplified and sequenced in both directions (*mutY*, *ureI*, *atpA*, *efp*, *ppa*, *trpC*, and *yphC*) [8, 49]. The genes were amplified by PCR using the conditions described by Achtman et al. [14]. The PCR products were purified using ExoSAP-IT® (Affymetrix, Cleveland, OH, USA) according to the manufacturer’s recommendations. The purified products were sequenced using the BigDye Terminator v3.1 Cycle Sequencing Kit in the ABI 3130 genetic analyser (Applied Biosystems, Foster City, CA, USA). Each strain was defined based on the presence of alleles for the seven genes (the allelic profile), and every allelic profile was defined as an ST [65, 66].

The datasets obtained in this article are available in the PubMLST database for *H. pylori* [http://pubmlst.org/helicobacter/] [41]. The accession numbers for each allele and ST are as follows: 2144, 2218, 2227, 2237, 2252, 2280, 2289, 2303, 2315, 2333, 2336, 2338, 2340, 2341, 2371, 2388, 2412, 2413, 2452, 2378, 2385, 2386, 2590, 2591, 2592, 2593, ST288, ST313, ST813, ST2887, ST2888, ST2889, ST2890, ST2891, ST2892, ST2893, ST2894, ST2895, ST2896, ST2897, ST2898, ST2899, and ST2900.

### Phylogenetic and genealogic analyses, genetic diversity analysis, and recombination

The sequences of the seven loci were aligned using ClustalX v2 [67], manually edited with Seaview v4.2.5 [68] and FinchTV V.1.4.0 software (Geospiza, Inc.), and compared with all known alleles of *H. pylori* deposited in the PubMLST database [http://pubmlst.org/helicobacter/]. To establish the open reading frame of the protein, the nucleotide sequences of different STs from each housekeeping gene were translated into amino acid sequences using the translate tool in ExPASy [http://www.expasy.org]. We used DnaSP v5.10 [69] to assess the nucleotide diversity, including the average nucleotide diversity per site (π) and the expected variation per site under the assumption of neutral evolution (θ), for each housekeeping gene. For phylogenetic analysis, the seven housekeeping genes from each strain were manually concatenated after their independent alignment.

The genealogic relationships among the *H. pylori* strains was inferred with the PHYLOViZ (http://www.phyloviz.net/) platform. PHYLOViZ infers evolutionary descent patterns among allelic profiles using the goeBURST algorithm and a full minimal spanning tree (MST)-like approach that uses a heuristic local optimization procedure [70]. The possible recombination events among the studied strains were explored with the neighbour-net algorithm [71] implemented in the SplitsTree4 program [72] using uncorrected P distances. The reliability of this network was confirmed with a non-parametric bootstrap analysis after 1000 pseudoreplications. Furthermore, the relatedness among the strains in this study and those deposited in the MLST database [http://pubmlst.org/helicobacter/] was determined using eBURST V3.0 [http://www.phyloviz.net/goeburst/]. This algorithm subdivides large MLST datasets into non-overlapping groups of related STs or clonal complexes to discern the location of the most parsimonious isolates within groups or clonal complexes based on the predicted founder. In addition, eBURST allows the observation of a “snapshot” population with a general view of the clonal complexes; the central part shows the main clonal complexes, the triple (TLV) and double (DLV) linkages, and the individual unlinked STs [65].

To determine the geographical type of *H. pylori* to which the strains analysed in this study belonged, a phylogenetic tree was generated with MEGA V6.0 software [26] using the neighbour-joining method and the Kimura 2-parameter model of nucleotide substitution [25, 73]. The concatenated nucleotide sequences of the seven housekeeping genes in the studied strains (17 ST), and reference strains (299) [http://pubmlst.org/helicobacter/], which were representative of different geographical groups, were aligned in Muscle software [74]. The reliability of clustering was evaluated with a non-parametric bootstrap test after 1000 pseudoreplications. The reference sequences of the geographical groups were as follows: hpEurope: 75 sequences, hpsNEAfrica: 14 sequences, hspWAfrica: 40 sequences, hspSAfrica: 50 sequences, hpAfrica2: 21 sequences, hspAmerind: 18 sequences, hspEastAsia: 30 sequences, hspMaori: 45 sequences, and hpAsia2: 6 sequences.

## Additional files

Additional file 1: **Table S1.** EPIYA motifs identified in the 80 paediatric strains of *Helicobacter pylori*. Nucleotide sequences of the EPIYA motifs identified in this study (XLSX 11 kb)

Additional file 2: **Table S2.** PubMLST accession numbers for the alleles and STs of *Helicobacter pylori* strains obtained from paediatric patients described in this study. *Alleles that are present in isolates from other global sources are indicated in red. List of PubMLST accession numbers for the alleles and STs identified in this study as well as alleles previously reported worldwide. (XLSX 11 kb)

## Data Availability

The datasets generated and/or analysed in the current study are available in the PubMLST database for *H. pylori* [http://pubmlst.org/helicobacter/]. Furthermore, the datasets supporting the conclusions of this article are included within the article and its supplementary tables.

## Abbreviations

AMX: Amoxicillin
ATCC: American Type Culture Collection
*atpA*: gene encoding an ATP synthase alpha chain
*babA*: gene encoding an outer membrane protein that binds to fucosylated Lewis b blood group antigen
*cagA*: gene encoding a cytotoxin-associated *gene A*
CLA: Clarithromycin
CLSI: Clinical and Laboratory Standards Institute
*efp*: gene encoding an elongation factor P
EPIYA: Glu-Pro-Ile-Tyr-Ala
MIC: Minimal Inhibitory Concentration
MLST: Multilocus Sequence Typing
MTZ: Metronidazole
*mutY*: gene encoding a DNA glycosylase
PCR: Polymerase Chain Reaction
*ppa*: gene encoding an inorganic pyrophosphatase
ST: Sequence Type
*trpC*: gene encoding an anthranilate isomerase
*ureI*: gene encoding a urease subunit I
*vacA*: gene encoding a vacuolating cytotoxin A
*yphC*: gene encoding a GTPase
